# Inflammatory Cell Interactions in the Rotator Cuff Microenvironment: Insights From Single-Cell Sequencing

**DOI:** 10.1155/ijog/6175946

**Published:** 2025-04-15

**Authors:** Wencai Liu, Xinyu Wang, Yuhao Yu, Weiming Lin, Hui Xu, Xiping Jiang, Chenrui Yuan, Yifei Wang, Xin Wang, Wei Song, Yaohua He

**Affiliations:** Department of Orthopaedics, Shanghai Sixth People's Hospital Affiliated to Shanghai Jiao Tong University School of Medicine, Shanghai, China

**Keywords:** cell interactions, inflammation, rotator cuff injury, single-cell RNA sequencing

## Abstract

Rotator cuff injuries are a common cause of shoulder pain and dysfunction, with chronic inflammation complicating recovery. Recent advances in single-cell RNA sequencing (scRNA-seq) have provided new insights into the immune cell interactions within the rotator cuff microenvironment during injury and healing. This review focuses on the application of scRNA-seq to explore the roles of immune and nonimmune cells, including macrophages, T-cells, fibroblasts, and myofibroblasts, in driving inflammation, tissue repair, and fibrosis. We discuss how immune cell crosstalk and interactions with the extracellular matrix influence the progression of healing or pathology. Single-cell analyses have identified distinct molecular signatures associated with chronic inflammation, which may contribute to persistent tissue damage. Additionally, we highlight the therapeutic potential of targeting inflammation in rotator cuff repair, emphasizing personalized medicine approaches. Overall, the integration of scRNA-seq in studying rotator cuff injuries enhances our understanding of the cellular mechanisms involved and offers new perspectives for developing targeted treatments in regenerative medicine.

## 1. Introduction

The human rotator cuff is a delicate and remarkable anatomical structure composed of four muscles and their extended tendons. This system not only supports shoulder mobility but also plays a critical role in maintaining the stability of the shoulder joint. Anatomically, the supraspinatus, infraspinatus, teres minor, and subscapularis muscles may seem independent, but they work in close harmony. These muscles, through precise coordination, firmly secure the humeral head within the glenoid fossa. This arrangement allows the shoulder to perform a wide range of complex movements, especially during abduction and external rotation, where they serve an essential and irreplaceable function [1]. Unfortunately, the rotator cuff is highly prone to injury. It can be damaged by sudden trauma, such as accidents, or through gradual wear and tear over time. Such injuries often result in a significant decline in shoulder function [2, 3]. Clinical data highlight a concerning trend: Rotator cuff tears are now a common health issue, especially among older adults. Studies show that nearly one-third of individuals over the age of 60 experience some form of rotator cuff problem. [4, 5] These injuries can range from minor fiber tears to complete ruptures, each causing varying levels of discomfort and disruption to daily life [6].

Rotator cuff injuries often result from repetitive overhead lifting, direct impacts, or the natural wear and tear associated with aging [7]. Activities such as swimming, baseball, and tennis, along with occupations that involve frequent overhead movements, significantly increase the risk of these injuries [8, 9]. As people age, their tendons become more prone to degeneration, making them more likely to tear even under minor stress [10]. Common symptoms of a rotator cuff injury include pain, loss of strength, reduced range of motion, and difficulty with overhead activities. These challenges can severely impact daily life and overall functional abilities [11].

Inflammation plays a critical role in the body's response to tissue damage and is essential for healing rotator cuff injuries [12]. This process occurs in two key phases: acute and chronic, both of which are vital for tendon repair and recovery. During the acute phase, immune cells gather at the injury site to clear debris, combat infection, and release signals that initiate tissue repair [13]. Macrophages, neutrophils, and other immune cells are instrumental in removing damaged cells and setting the stage for the healing process [14].

While inflammation is essential for healing, unresolved or prolonged inflammation can progress into chronic inflammation, which is often linked to musculoskeletal conditions like rotator cuff disorders. Chronic inflammation in the rotator cuff tendon can disrupt the healing process, accelerate tissue degeneration, and contribute to the development of fibrosis [15, 16]. This shift from an acute, restorative phase to a chronic, harmful phase is driven by persistent inflammatory agents, such as cytokines and growth factors, which promote fibrosis and tissue necrosis and interfere with collagen synthesis [17, 18]. Key players in this process include macrophages, T-cells, and fibroblasts, which influence both tissue repair and degeneration.

This paper focuses on the role of immune cell interactions in rotator cuff injuries, particularly how inflammation affects both healing and the progression to chronic disease. Advances in single-cell sequencing technology have provided unprecedented insights into the complex cellular landscape of inflammatory responses. These tools allow researchers to identify specific subsets of immune and nonimmune cells, analyze their gene expression profiles, and study their dynamic interactions at the injury site [19, 20]. Applying these technologies to rotator cuff injuries has revealed intricate cellular networks that regulate tendon repair and the transition to chronic inflammation.

The review highlights the transformative impact of single-cell RNA sequencing (scRNA-seq) and related methods, such as spatial transcriptomics, in understanding immune cell behavior within the rotator cuff microenvironment at a single-cell resolution. By exploring the roles of critical cellular components—macrophages, T-cells, and fibroblasts, this review aims to deepen our understanding of the molecular processes involved in tendon healing. Additionally, it examines how these findings can inform therapeutic strategies to mitigate chronic inflammation, promote effective tendon repair, and prevent long-term functional impairments.

## 2. Single-Cell Sequencing Technologies in Inflammation Research

### 2.1. Overview of scRNA-seq

**Figure 1:**
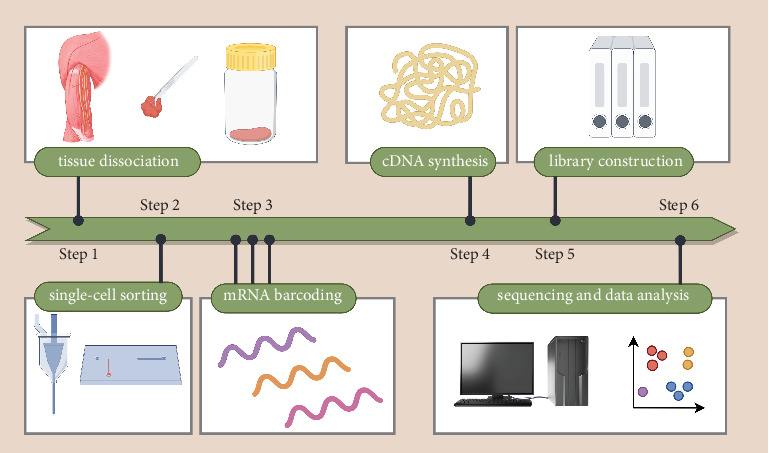
Single-cell RNA sequencing (scRNA-seq) workflow. The diagram outlines the scRNA-seq process: tissue dissociation, single-cell sorting, mRNA barcoding, cDNA synthesis, library construction, sequencing, and data analysis, facilitating the exploration of cellular diversity and transcriptional profiles.

scRNA-seq has revolutionized research by providing detailed insights into the unique characteristics of individual cells within complex tissues (Figure 1). It allows for a precise analysis of each cell's genetic expression, offering invaluable information about how different cell type functions. This level of granularity is particularly useful for studying the rotator cuff, where both immune and nonimmune cells are highly active during inflammation, injury, and the healing process.

Recent advancements in complementary technologies, such as spatial transcriptomics and multiomics, have further enhanced the power of scRNA-seq. These innovations allow researchers to explore tissue environments in even greater detail. Spatial transcriptomics, for example, integrates gene expression data with spatial information, enabling scientists to identify not only what is happening within individual cells but also where these cells are located within the tissue and how they interact [21, 22]. This spatial context is critical in the rotator cuff, where the position and organization of cells significantly influence how the tissue responds to damage and initiates repair. Furthermore, single-cell multiomics approaches, such as CITE-Seq, have identified discrete tendon cell populations that persist in vitro, highlighting specific gene and surface-protein signatures that are crucial for tendon disease modeling and therapy development [23]. Additionally, multiomics approaches, which examine multiple biological layers such as DNA, RNA, and proteins, provide a more comprehensive understanding of cellular functions and regulatory mechanisms.

Advancements in technology have significantly expanded our understanding of cellular diversity, offering deeper insights into the roles various cell types play in biological processes [24]. scRNA-seq helps identify different cell types and reveals how they interact in both healthy and diseased conditions. [25, 26] In the context of the rotator cuff, this technology can illuminate the intricate interactions among macrophages, T-cells, fibroblasts, and other cell types during inflammation and tissue repair. By tracking these changes over time and across different tissue regions, scRNA-seq enables the creation of detailed cellular maps, identifying critical regulatory nodes that influence cell fate and differentiation. These discoveries are essential for understanding the molecular mechanisms underlying tissue growth, healing, and degeneration [27].

Beyond its applications in inflammation and tissue repair, scRNA-seq has also made significant contributions to cancer research, where it has been used to analyze the complex cellular environments of tumors. By mapping interactions between tumor cells, immune cells, and stromal cells, scRNA-seq has not only provided insights into tumor heterogeneity and drug resistance but also opened new avenues for treatment development [26, 28, 29]. Applying this technology to chronic rotator cuff injuries offers the potential to unravel how immune and stromal cells contribute to inflammation and healing failures, paving the way for novel therapeutic strategies.

With continuous advancements and decreasing costs, scRNA-seq is becoming increasingly accessible and widely used in biomedical research. This progress holds promise for improving disease diagnosis and enabling precision medicine [30]. For rotator cuff disorders, integrating scRNA-seq with high-throughput methods and big data analysis could revolutionize personalized diagnostic and therapeutic approaches. Ultimately, scRNA-seq is poised to transform our understanding of the cellular environment in the rotator cuff; providing fresh insights into inflammation, tissue repair, and disease progression; and driving the development of targeted, effective interventions.

### 2.2. Application in Inflammation

scRNA-seq technology has revolutionized inflammation research, allowing scientists to examine cellular responses in unprecedented detail. Acting as a magnifying lens, it uncovers functional changes, regulatory networks, and unique cell characteristics. This powerful tool provides insights into inflammation at the molecular level, helping researchers understand tissue damage and repair mechanisms [31–35].

scRNA-seq has also transformed our understanding of immune cell interactions in inflammatory conditions. For example, studies on lung fibrosis have used this technology to identify key cell populations, map genetic profiles, and trace intercellular communication pathways involved in disease progression. [35] These findings highlight the critical role of inflammatory cells in shaping disease outcomes.

Additionally, scRNA-seq is indispensable for identifying immune cell subpopulations. In tumor inflammatory microenvironments, it has revealed distinct immune cell subsets with unique traits that influence disease progression and serve as therapeutic targets [32, 33]. Using cell type-specific markers, researchers can classify immune phenotypes, advancing personalized therapeutic strategies and refining disease categorization.

In chronic inflammatory diseases, integrating scRNA-seq with functional genomics has provided insights into stromal cell roles in sustaining inflammation and driving disease progression [34]. Stromal cells interact closely with immune cells through specific signaling pathways, influencing inflammatory states. Single-cell analyses have identified stromal subpopulations linked to fibrosis and tissue remodeling, revealing key contributors to maladaptive repair processes. [34]

Advancements in multiomics approaches have further expanded scRNA-seq's potential. By combining genomic, transcriptomic, and epigenomic analyses, researchers can dissect complex inflammatory models and uncover previously unrecognized cellular diversity [31, 33]. Investigating inflammation at the single-cell level not only deepens molecular understanding but also paves the way for personalized therapies.

Overall, scRNA-seq is a powerful tool for unraveling the molecular mechanisms of inflammation. By clarifying immune–stromal interactions, it provides crucial insights into disease pathophysiology. These findings are essential for developing targeted therapies for inflammatory disorders and tissue injuries, including rotator cuff damage.

### 2.3. Advances in Spatial Transcriptomics and Spatial Proteomics in Inflammation Research

Recent advancements in spatial transcriptomics and spatial proteomics have significantly enhanced the understanding of cellular heterogeneity and tissue organization, particularly in the context of inflammation and tissue repair in musculoskeletal injuries such as tendinopathy. These technologies complement single-cell sequencing by providing spatial context to gene and protein expressions, which is crucial for understanding complex tissue dynamics. The integration of spatial and single-cell technologies offers a comprehensive view of cellular interactions and molecular mechanisms underlying tissue repair processes.

Spatial transcriptomics technologies have evolved to provide high-resolution maps of gene expression within tissues, preserving spatial information that is lost in traditional single-cell RNA sequencing. This allows for the identification of spatially colocalized cell populations and the dynamics of gene expression in situ [36, 37]. Methods such as expansion sequencing (ExSeq) combine in situ sequencing with expansion microscopy, enhancing the resolution of RNA transcripts to the nanoscale level. This allows for detailed exploration of cellular RNA in both targeted and untargeted approaches, providing insights into cellular identity and interactions [38]. Techniques like Deep-STARmap and Deep-RIBOmap enable 3D in situ quantification of gene transcripts and translation activities within thick tissue blocks, facilitating comprehensive analysis of tissue structure and function in health and disease [39].

Spatial proteomics provides visualization and quantification of protein expression profiles at single-cell resolution, offering detailed spatial context within tissues. This includes the spatial composition of cell types and the distribution of functional structures, which are crucial for understanding cellular distribution patterns [40]. The development of resources like scProAtlas provides comprehensive spatial functional annotation, aiding in the understanding of spatial structures and interactions within various tissue types [40].

The combination of spatial transcriptomics and scRNA-seq allows for the creation of detailed cell atlases, revealing cellular heterogeneity and molecular mechanisms in conditions like tendinopathy. This integration helps identify specific cell subtypes and their spatial distributions, which are critical for understanding disease progression and tissue repair [41, 42]. Spatial technologies enable the analysis of cell–cell interactions within their native tissue context, providing insights into the dynamic cellular environment that drives disease development. This is particularly useful in identifying potential therapeutic targets in inflammatory and degenerative conditions [41].

While these advancements offer significant insights, it is important to consider the limitations and challenges associated with these technologies. Spatial transcriptomics and proteomics are still evolving, with ongoing efforts to improve resolution, throughput, and integration with other omics data. Additionally, the variability across different platforms and the need for standardized evaluation metrics remain challenges that need to be addressed to fully realize the potential of these technologies in biomedical research [43, 44].

## 3. Immune Cells in the Rotator Cuff Microenvironment

### 3.1. Macrophages

Macrophages are key regulators of the rotator cuff microenvironment, controlling inflammation and tissue repair through various functions. The advent of scRNA-seq has revolutionized our understanding of macrophage diversity, revealing distinct subpopulations with specific roles in tissue repair, inflammation, and scarring. In rotator cuff injuries, scRNA-seq has uncovered significant differences in macrophage gene expression, particularly between degenerative tears and acute trauma [45]. This diversity is critical for deciphering how macrophages influence varied healing trajectories.

In tendon repair, macrophages catalyze essential processes, including wound debridement, extracellular matrix (ECM) deposition, and fibroblast proliferation—all crucial for tissue regeneration. Tendon-resident macrophages have emerged as key players in these stages, highlighting their specialized roles. The chemokine receptor CCR2 is essential for recruiting macrophages to injury sites. The absence of CCR2 impairs macrophage recruitment, reduces myofibroblast activity, and delays ECM reconstruction, thereby hindering tendon healing [46]. This underscores the importance of targeted macrophage homing in rotator cuff repair.

Further exploration of macrophage subpopulations in musculoskeletal injuries has identified specific transcriptional markers linked to either regenerative or fibrotic healing pathways. For instance, regenerative niches exhibit enhanced platelet-derived growth factor (PDGF) signaling, promoting cellular proliferation and tissue restoration. Conversely, fibrotic niches display increased transforming growth factor beta (TGF-*β*) activity, driving fibroblast activation and excessive ECM deposition [47]. These molecular markers highlight the pivotal role of macrophage-mediated signaling in determining whether the wound environment heals effectively or progresses to chronic pathology.

In muscle regeneration, certain macrophage subtypes, such as those expressing GPNMB, play critical roles in promoting repair, emphasizing the therapeutic potential of targeting macrophage-specific pathways [48]. This finding may extend to rotator cuff injuries, where macrophage dysfunction or misregulation correlates with prolonged healing or fibrotic scarring. Additionally, macrophages in fibrotic conditions, such as pulmonary fibrosis, exhibit unique gene expression patterns that enhance fibroblast activity and pathological ECM remodeling, providing a comparative model for understanding macrophage behavior in rotator cuff injuries [49].

Recent studies emphasize the critical role of macrophage polarization in tendon healing. Various approaches have been developed to promote M2 macrophage polarization and enhance rotator cuff repair, including inflammation-responsive hydrogels releasing SDF-1 and IL-4, dendritic cell-derived exosomes (DEXs), and 3D-printed Mg-incorporated scaffolds [50–52]. Additionally, electrospun nanoyarn loaded with naproxen sodium accelerates M2 polarization, while cyclooxygenase (COX) regulation via the Pla1a/Etv1 axis reduces adhesion formation and strengthens tendons [53, 54]. These strategies highlight macrophage-targeted therapies as promising avenues for improving tendon regeneration and inflammation resolution.

### 3.2. T-Cells

T-cells occupy a central role in the complex ecosystem of rotator cuff injuries, driving both inflammation and tissue reconstruction. Their interactions with tenocytes are particularly significant, forming a feedback loop that intensifies inflammation and shifts the collagen composition toward a detrimental collagen type III/type I imbalance. This interaction is driven by increased inflammatory cytokines and chemokines secreted by tenocytes, which activate T-cells and further perpetuate the inflammatory cycle [55].

scRNA-seq has significantly advanced our understanding of the diversity and functions of various T-cell populations in tendon injuries. By identifying distinct T-cell subtypes and their genetic profiles, scRNA-seq has deepened our insights into T-cell adaptability and their responses to tissue damage [56]. In rotator cuff injuries, regulatory T-cells (Tregs) are observed to proliferate, acting as a control mechanism to mitigate excessive immune responses and potentially reduce tissue damage [57].

The migration of specific T-cell subsets, including T helper (Th) cells and Tregs, into injured muscle and tendon highlights their roles in both inflammation and repair. Th cells are primary drivers of inflammatory responses, while Tregs are believed to suppress excessive inflammation and facilitate tissue repair, underscoring the critical balance of these cells in the healing process [58].

These findings emphasize the indispensable role of T-cells in the onset and resolution of rotator cuff injuries. Modulating T-cell-mediated pathways—by enhancing Treg activity or suppressing harmful Th responses—could present a transformative therapeutic strategy for improving tendon repair [59]. Ultimately, scRNA-seq has been instrumental in elucidating the multifaceted roles of T-cells within tendon microenvironments, paving the way for tailored therapies that finely tune immune responses in rotator cuff injuries.

### 3.3. Fibroblasts and Myofibroblasts

In the intricate microenvironment of the rotator cuff, fibroblasts and myofibroblasts play vital roles in orchestrating fibrosis and tissue remodeling. The wealth of data derived from scRNA-seq has been instrumental in uncovering the diverse functions and pivotal roles of these cells. Fibroblasts serve as central players in ECM deposition and remodeling, while their activated counterparts, myofibroblasts, not only facilitate tissue repair but also drive fibrosis, contributing to both healing and scar formation [60].

Using scRNA-seq, the transcriptional and functional heterogeneity of fibroblasts has been delineated, identifying distinct subpopulations involved in tissue remodeling and fibrosis. For instance, in the rotator cuff, scRNA-seq has enabled the mapping of the shoulder synovial environment, identifying fibroblast subsets with specific roles in ECM remodeling, inflammation regulation, and cell proliferation [61]. These findings illuminate how fibroblasts respond to microenvironmental cues, contributing to both repair and pathological transformations.

Additionally, scRNA-seq has provided insights into the fibrocartilaginous enthesis of the rotator cuff, revealing molecular intricacies associated with fibrocartilage differentiation. This transcriptional analysis serves as an invaluable tool for understanding the growth and degeneration of the enthesis, particularly under disease-related conditions [62].

Moreover, scRNA-seq has highlighted the dual role of mesenchymal stromal cells (MSCs) in fibrosis, showing that MSCs can either promote or inhibit fibrosis through their differentiation into myofibroblasts. Genes such as neurotrimin and CHD3 have been implicated in these regulatory processes, identifying potential molecular targets for modulating fibrosis progression [63].

Collectively, these findings underscore the complex roles of fibroblasts and myofibroblasts in fibrosis and tissue remodeling. scRNA-seq continues to be an invaluable tool for elucidating the diversity within these cell types, providing fresh perspectives that could inform innovative therapeutic strategies to balance repair and scar formation in rotator cuff injuries [64].

### 3.4. Other Immune and Nonimmune Cells

In the complex ecosystem of the rotator cuff, various cells, beyond just immune ones, play pivotal roles in both inflammatory responses and the healing process, as shown by recent single-cell sequencing studies. Immune system key players, such as B cells, natural killer cells, and dendritic cells, are crucial in addressing musculoskeletal injuries like rotator cuff damage. Alongside macrophages and T cells, these immune cells not only sustain the inflammatory environment but also actively participate in reconstructing and repairing damaged tissue [19, 65]. Advanced scRNA-seq has further highlighted the intricate interactions between immune cells and supportive stromal cells, such as mesenchymal progenitor cells (MPCs), which modulate immune responses and facilitate healing through diverse receptor-ligand mechanisms [65]. Nonimmune cells, including endothelial cells, adipocytes, and smooth muscle cells, also significantly influence the rotator cuff's internal environment. For instance, endothelial cells interact with perivascular cells, which resemble stem cells, potentially aiding tissue repair and fibrosis via pathways associated with ECM proteins [41]. Adipocytes and fibroblasts contribute to fatty infiltration and fibrosis, commonly observed after rotator cuff tears, with their functions regulated by immune cell infiltration [66]. Combining single-cell sequencing with spatial transcriptomics reveals how cells interact, identifies immune imbalances in tendon injuries, and suggests new therapies for rotator cuff healing [41].

## 4. Molecular Pathways Driving Inflammation and Healing

### 4.1. Cytokine and Chemokine Networks

The sophisticated methodology of single-cell sequencing has revealed the complex interactions of cytokines and chemokines within the microenvironment of the rotator cuff, underscoring their pivotal roles in modulating inflammatory responses and tissue repair. Macrophages and T cells are central to these interactions, with the chemokine receptor CCR2 emerging as a critical mediator of macrophage migration to injury sites. Both resident tendon macrophages and T cells express this receptor, and its absence impairs the healing process by reducing the numbers of macrophages and myofibroblasts, which are essential for functional recovery [67].

The diversity within macrophages, particularly the proinflammatory M1 subset, and T cells is significant, as these cells perform distinct functions in controlling inflammation and facilitating repair. Moreover, cytokines such as IL-1*β*, TNF*α*, and IL-6, along with chemokines like CCL2, are crucial for maintaining ECM homeostasis and remodeling, which are critical steps in tendon repair [68]. Dysregulation of these signaling molecules can lead to tissue disarray, thereby delaying recovery.

At the molecular level, NF-*κ*B signaling pathways, particularly through IKK*β*, play a central role in inflamm-aging and the senescence of tendon-derived stem/progenitor cells. This suggests that targeting NF-*κ*B signaling therapeutically could rejuvenate senescent cells, restoring their regenerative capacity and improving healing outcomes [69]. Similarly, the activation of the NLRP3 inflammasome pathway by danger signals such as high mobility group box 1 (HMGB1) has been linked to inflammatory responses and ECM disruption in injured rotator cuff tendons [70].

In summary, these findings highlight the dynamic and highly coordinated immune response mediated by cytokines, chemokines, and signaling pathways, which govern tendon inflammation and repair. Targeting these pathways presents a promising approach for developing therapeutic strategies to modulate the immune microenvironment, optimize ECM remodeling, and ultimately enhance rotator cuff healing outcomes.

### 4.2. Signaling Pathways

Single-cell sequencing has significantly advanced the understanding of signaling pathways that regulate inflammation and tissue repair in rotator cuff injuries. Among these pathways, AMPK, a metabolic checkpoint, and TREM-1, an inflammatory mediator, are notably upregulated in injuries associated with fatty infiltration and inflammation [71]. These pathways underscore the intricate relationship between metabolic regulation and inflammation in tendon pathology.

scRNA-seq has revealed the diversity of cell types involved in these pathways, identifying unique subpopulations that drive both inflammatory and healing processes [72]. For example, satellite cells have been shown to produce inflammatory signaling molecules such as TNF-*α*, CCL2, and IL-6, which not only attract immune cells to the injury site but also stimulate their proliferation, contributing to tissue regeneration [73].

Additionally, scRNA-seq has identified novel macrophage subtypes and genes like RAB38, which regulate osteoclast precursor cells and facilitate bone healing, further illuminating the cellular dynamics underlying musculoskeletal repair [74].

These findings highlight the immense potential of single-cell technologies in decoding complex signaling pathways, offering new avenues for therapeutic interventions aimed at enhancing tissue regeneration and recovery in rotator cuff injuries.

### 4.3. Tissue Remodeling and Fibrosis

The use of single-cell sequencing has led to transformative discoveries in the repair and fibrosis of rotator cuff tissue, shedding light on the intricate mechanisms underpinning the healing process. In the microenvironment of the rotator cuff enthesis, scRNA-seq has played a critical role in tracing the maturation and specialization of fibrocartilage postnatally. This technique unveils the dynamic cellular distribution and transcriptional changes associated with tissue maturation and repair [62]. Such detailed insights are vital for identifying key cell types and understanding their specific roles in tissue regeneration and fibrosis, forming a foundation for the development of precise therapeutic strategies.

Particularly in frozen shoulder, a condition characterized by inflammatory fibrosis, studies have identified specialized macrophage populations that contribute to resolving inflammation. Notably, the interaction between MERTK+ macrophages and DKK3+ or POSTN+ fibroblasts outlines a cellular framework for fibrosis resolution, presenting new therapeutic targets to promote tissue repair [75, 76].

Moreover, the integration of spatial transcriptomics has deepened our understanding of regional cellular activity variations in muscle tissues affected by rotator cuff tears. This approach highlights the heterogeneous nature of muscle pathology and identifies novel transcriptional pathways with therapeutic potential [77].

Taken together, single-cell and spatial transcriptomic technologies hold exceptional promise for unraveling the cellular and molecular dynamics of tissue remodeling and fibrosis. By elucidating these processes, these advanced tools pave the way for targeted therapies that can enhance recovery in musculoskeletal disorders such as rotator cuff injuries [78].

## 5. Immune–Nonimmune Cell Interactions in Rotator Cuff Injury

### 5.1. Crosstalk Between Macrophages and Fibroblasts

The interactions between macrophages and fibroblasts are critical in regulating the balance between tendon healing and fibrosis in rotator cuff injuries. The polarization states of macrophages play a decisive role in this process: M1 macrophages drive proinflammatory responses that can worsen fibrosis, whereas M2 macrophages support anti-inflammatory pathways and promote tissue regeneration [79, 80]. Encouraging a shift from the M1 to the M2 phenotype—achievable through interventions like disulfiram—reduces the secretion of proinflammatory cytokines, thereby suppressing fibroblast activity, alleviating fibrosis, and enhancing tendon repair [80].

A pivotal mechanism involves macrophage-derived exosomes, especially those containing miR-21-5p. These exosomes trigger fibrosis by targeting pathways such as Smad7, a key regulator of fibrogenesis [81]. Additionally, the recruitment of macrophages to the injury site, mediated by chemokines like CCL2 and their receptors such as CCR2, influences fibroblast activation and the overall fibrosis. Importantly, temporally regulated antagonism of CCR2 can fine-tune macrophage recruitment, fostering a more favorable inflammatory environment and improving tendon healing outcomes [82]. Moreover, the intricate interplay between macrophages and fibroblasts is further shaped by T-cell–mediated responses, adding another layer of complexity to immune regulation in tendon healing. These interactions can either exacerbate or resolve fibrosis, depending on the immune microenvironment and timing of intervention [83].

Single-cell sequencing has deepened our understanding of macrophage–fibroblast interactions by identifying critical pathways and cellular dynamics during tendon repair. In particular, resident macrophages expressing CD206 interact closely with fibroblasts and the ECM, regulating fibroblast phenotype and ECM composition. These macrophages exhibit the expression of various ECM-related genes, underscoring their supportive role in tendon growth and repair [84].

In injury contexts, scRNA-seq has uncovered specific macrophage subpopulations that localize to fibroblast-accumulated regions and exhibit a profibrotic phenotype, as seen in lung fibrosis models. These macrophages, characterized by high expression of Cx3cr1 and MHCII, demonstrate a trophic influence on fibroblast activation, suggesting a similar role in rotator cuff repair [49].

Further evidence highlights the role of tendon stem cell-derived exosomes in modulating macrophage polarization. These exosomes transition macrophages from an inflammatory M1 type to an anti-inflammatory M2 variety, creating a conducive regenerative setting and improving the efficacy of tendon-to-bone repair [85]. Additionally, extracellular vesicle-educated macrophages have demonstrated promising effects in early tendon healing by mitigating inflammation and enhancing mechanical properties. This highlights the therapeutic potential of macrophage polarization in facilitating tendon repair [86].

In conclusion, macrophage–fibroblast interactions serve as a critical axis in tendon healing and fibrosis. Single-cell technologies have provided valuable insights into the intricate signaling networks and cellular behaviors driving this process. Targeting macrophage activity and polarization offers significant promise for developing therapies aimed at improving tendon healing and minimizing fibrotic complications in rotator cuff injuries.

### 5.2. Immune Cell–Vascular Interactions

**Figure 2:**
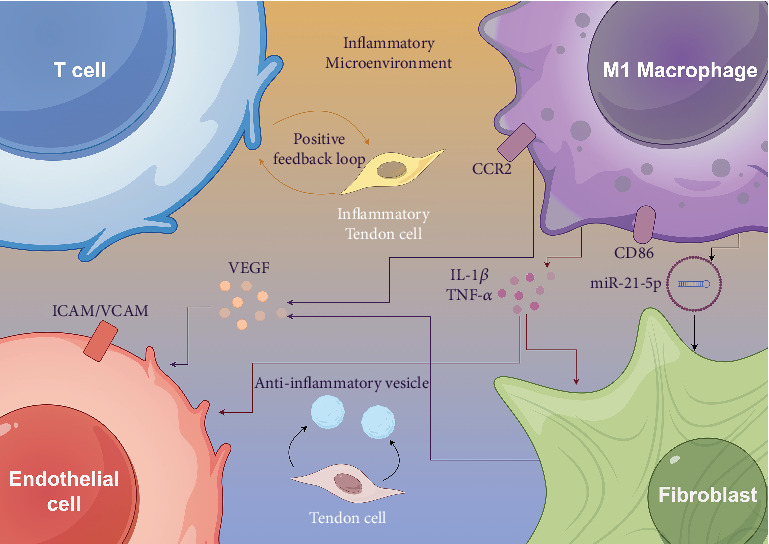
Cellular interactions in the inflammatory microenvironment of the rotator cuff. This schematic illustrates the complex interplay between various cell types within the inflamed rotator cuff microenvironment, including immune cells (e.g., macrophages and T-cells), fibroblasts, and endothelial cells. The diagram highlights the signaling pathways and cytokine exchanges that contribute to inflammation, tissue remodeling, and healing processes following rotator cuff injury.

Interactions between the vascular and immune systems are fundamental to both the development and recovery of rotator cuff injuries (Figure 2). The infiltration and activation of immune cells, particularly macrophages, significantly influence tissue degeneration and repair. Following injury, there is a notable influx of mononuclear phagocytes, such as unconventional macrophage subtypes, underscoring their roles in tissue reconstruction and inflammation resolution [58]. Cytokines associated with angiogenesis, such as vascular endothelial growth factor (VEGF), are elevated in rotator cuff disorders and correlate with tendon degeneration and vascular remodeling [87]. Moreover, lymphangiogenesis, the formation of new lymphatic vessels, supports rotator cuff healing by modulating inflammation and promoting tissue repair, as evidenced by the detrimental effects of lymphatic suppression on healing outcomes [88].

The immune system's pivotal role is further highlighted by the involvement of diverse immune cell types, such as T cells and macrophages, which coordinate with stromal cells to mediate tissue degradation and remodeling [59]. Macrophage polarization significantly shapes the inflammatory environment and healing processes [89]. The interplay between immune cells and vascular elements, including cytokines and lymphatic vessels, underscores the complexity of immune responses in rotator cuff injuries and reveals therapeutic opportunities for enhancing repair and recovery by modulating these pathways. [65, 90, 91]

scRNA-seq has provided critical insights into the interactions between the vascular and immune systems within the rotator cuff microenvironment. This advanced technology enables detailed profiling of cellular heterogeneity, which often goes unnoticed with bulk sequencing methods, thereby uncovering novel cell types and states that contribute to the immune microenvironment's roles in tissue development, homeostasis, and disease [20, 92]. Notably, scRNA-seq has been instrumental in identifying cell–cell communication networks, such as ICAM and VCAM signaling pathways, that govern interactions between immune cells and endothelial cells [93]. These interactions are not only critical in pathological conditions, such as intravascular lymphomas, but also in understanding immune responses during tissue repair and regeneration, as seen in rotator cuff injuries. Mapping these interactions at a single-cell level offers a comprehensive understanding of the immune–vascular microenvironment and its implications for therapeutic strategies [20].

### 5.3. Cellular Communication and Niche Factors

ECM and niche factors play a key role in regulating immune cell behavior within the rotator cuff microenvironment. The ECM is more than just a structural scaffold; it provides biochemical signals that influence immune cell migration, adhesion, and function. During inflammation, ECM components interact with cytokines and enzymes to coordinate leukocyte recruitment and effector activities at the injury site [94, 95]. In the rotator cuff, ECM remodeling affects the immune microenvironment and the healing process. For instance, engineered stem cell niche matrices have been shown to enhance tendon regeneration by modulating local immune responses. This demonstrates the importance of ECM-mediated autocrine and paracrine signaling [96]. Research on muscle satellite cell niches has also highlighted how the ECM regulates cell localization, activation, and differentiation—processes essential for tissue repair [97]. Furthermore, ECM-derived peptides mimic these regulatory effects, promoting cell proliferation and differentiation to support regeneration [98].

Advances in single-cell spatial transcriptomics have deepened our understanding of cellular organization and communication in tendon and rotator cuff healing. In tendinopathy, spatial mapping has identified distinct phenotypes of endothelial, stromal, and immune cells. Notably, mural cells show progenitor-like roles similar to those seen in rheumatoid arthritis. These interactions influence fibroblast differentiation and ECM production, which are central to pathological tissue remodeling [41].

T-cell–tenocyte interactions create a self-sustaining inflammatory loop that disrupts collagen composition, emphasizing the role of immune-stromal crosstalk in chronic tendon disease [55]. Spatial transcriptomics has also revealed tenocyte subpopulation heterogeneity and their spatial distribution. This provides valuable insights into how inflammatory infiltration and tissue degeneration progress in tendinopathy [42].

In rotator cuff healing, spatial transcriptomics has highlighted regional patterns of muscle fiber degeneration and regeneration. These patterns reflect niche-specific cellular activities [77]. Together, these findings emphasize the critical role of intercellular signaling and cellular niches in balancing inflammation, tissue restoration, and breakdown. They provide a clearer understanding of the complex processes involved in rotator cuff disorders and their healing mechanisms.

### 5.4. Molecular Signatures of Immune and Fibroblast Activity in Tendon Healing and Fibrosis

The gene expression signatures in macrophages, T-cells, and fibroblasts play a crucial role in regulating the balance between tendon healing and fibrosis in rotator cuff injuries. Understanding these molecular signatures provides critical insights into potential therapeutic targets for improving tendon repair and reducing fibrosis.

Macrophages exhibit distinct gene expression profiles that influence their role in the healing process. One key factor is CCR2, a chemokine receptor that mediates macrophage recruitment to injury sites. Tendon-resident macrophages and T-cells express CCR2, and its absence has been shown to impair tendon healing, demonstrating its essential role in macrophage-mediated repair processes [46]. Additionally, SPP1+ macrophages are linked to fibrotic tendon adhesion, whereas FOLR2+ macrophages form an antifibrotic cluster. The migration of FOLR2+ macrophages is regulated by ACKR1, highlighting a potential therapeutic target for reducing fibrosis [99]. Furthermore, macrophages under mechanical stimulation can polarize to the M2 phenotype, promoting transforming growth factor-beta 1 (TGF-*β*1) production. This cytokine plays a vital role in mesenchymal stem cell (MSC) chondrogenesis, ultimately enhancing tendon–bone healing [100].

T-cells also display gene expression heterogeneity that affects the inflammatory response and healing process. The presence of CCR2+ T-cells suggests that T-cells participate in immune regulation and tissue repair [67, 101]. However, their role in chronic inflammation must be tightly controlled, as persistent T-cell-driven inflammatory responses can shift the balance toward fibrotic tissue formation [101].

Fibroblasts contribute to tendon healing and fibrosis through distinct gene expression patterns. ADAM12+ fibroblasts are associated with tendon adhesion formation, indicating their involvement in fibrotic remodeling [99]. Moreover, fibroblasts express high levels of Csf1, the gene encoding macrophage colony-stimulating factor (M-CSF). This signaling molecule facilitates macrophage–fibroblast interactions, which are crucial for ECM regulation and fibroblast function in tendon repair [84].

Given the complexity of immune and fibroblast interactions in tendon healing, it is evident that targeting a single molecular pathway may not be sufficient. The balance between proinflammatory and anti-inflammatory macrophages, fibroblast-driven ECM remodeling, and T-cell-mediated immune regulation determines whether the tendon heals effectively or progresses toward fibrosis. Therefore, developing multitargeted therapeutic approaches that fine-tune these interactions could lead to improved regenerative outcomes while minimizing fibrosis.

## 6. Chronic Inflammation and Progression to Pathology

### 6.1. Factors Leading to Chronic Inflammation

Chronic inflammation in rotator cuff injuries is sustained by multiple mechanisms that hinder resolution and contribute to ongoing pathology. Tendon cells exhibit dysregulated resolution responses, marked by elevated levels of both specialized proresolving mediators (SPMs) and inflammation-initiating eicosanoids. This imbalance, where SPMs fail to counteract persistent inflammation, underpins the development of chronic conditions [102]. Additionally, a self-reinforcing cycle exists between T cells and tenocytes, further intensifying the inflammatory response. Once activated, tenocytes increase their production of inflammatory cytokines and chemokines, attracting and stimulating more T cells. In turn, T cell activation heightens the release of inflammatory mediators by tenocytes, disrupting the collagen matrix and perpetuating tissue damage [55]. The inability to resolve inflammation is further exacerbated by impaired repair mechanisms and excessive inflammatory responses, both of which are hallmarks of chronic diseases [103, 104].

Although inflammation is necessary for clearing debris and initiating tissue repair, its prolonged persistence in tendon injuries leads to chronic tendinopathy. This condition is characterized by tissue degeneration, regeneration, and microtears in the absence of acute inflammation [105, 106]. The inflammatory landscape in tendon injuries is further complicated by the interaction of proinflammatory agents, including cytokines and neuropeptides. This intricate network blurs the line between chemical and neurogenic inflammation, adding another layer of complexity to chronic tendon pathology [105].

### 6.2. Single-Cell Insights Into Chronic Inflammatory Signatures

Single-cell sequencing has emerged as an essential tool for identifying changes in immune cell composition, revealing gene expression patterns, and discovering potential biomarkers associated with chronic inflammation and fibrosis in rotator cuff disorders. This technology enables detailed characterization of cellular phenotypes and interactions within diseased tissues, offering new insights into the pathogenesis of tendon disorders. Techniques such as scRNA-seq and spatial transcriptomics have provided a comprehensive map of the cellular landscape in these conditions. These approaches have identified various immune and connective tissue cells with altered genetic activity, including increased expression of ECM protein genes—an indicator of tendinopathy [42]. In frozen shoulder, a chronic inflammatory fibrotic condition, single-cell analysis has identified distinct macrophage populations, including MERTKlowCD48+ and MERTK+LYVE1+MRC1+ macrophages. These cells play key roles in regulating inflammation and resolving fibrosis. Their interactions with fibroblasts suggest the mechanisms for matrix remodeling and disease resolution [76]. Advanced tools like ImmunIC have further enhanced the precision of identifying immune cells within single-cell datasets. This innovation is crucial for uncovering disease-specific immune markers and exploring potential therapeutic targets [107]. Moreover, single-cell genomics is providing new perspectives on the complex immune landscapes in chronic inflammatory conditions. It meticulously documents immune cell responses and their interactions across different tissues [108]. Collectively, these findings underscore the indispensable role of single-cell sequencing in understanding the cellular and molecular mechanisms underlying chronic inflammation and fibrosis in rotator cuff disorders. This progress paves the way for more precise and targeted therapeutic strategies.

### 6.3. Cytokine and Chemokine Networks in Chronic Rotator Cuff Inflammation

Chronic inflammation in rotator cuff injuries is sustained by a complex interplay of cytokines, chemokines, and signaling pathways, which contribute to fibrosis and impaired healing. Key players in this process include the NF-*κ*B signaling pathway, the receptor for advanced glycation end-products (RAGE), and various inflammatory cytokines such as TNF-*α* and IL-1*β*. These elements not only perpetuate inflammation but also promote fibrotic changes that hinder effective tissue repair.

TNF-*α* and IL-1*β* are among the most significant proinflammatory cytokines upregulated in rotator cuff injuries, where they contribute to ECM disorganization and persistent inflammation. TNF-*α*, in particular, plays a central role in activating the NF-*κ*B pathway, exacerbating inflammatory responses and accelerating tendon degeneration [70, 109]. Another key inflammatory mediator is HMGB1, a damage-associated molecular pattern (DAMP) protein that is elevated in chronic rotator cuff injuries. HMGB1 sustains inflammation by activating the NLRP3 inflammasome, further driving the release of inflammatory cytokines and perpetuating tissue damage [70, 110]. Additionally, TGF-*β*1 is a well-established driver of fibrosis, promoting myofibroblast differentiation and excessive ECM deposition, both of which contribute to the fibrotic changes seen in chronic tendon injuries [111].

The NF-*κ*B signaling pathway is a primary mediator of chronic inflammation in rotator cuff injuries. Activated by TNF-*α* and other inflammatory mediators, NF-*κ*B regulates the transcription of multiple proinflammatory genes, exacerbating tissue damage. Research suggests that inhibition of NF-*κ*B signaling can reduce inflammation and improve healing outcomes in tendon injuries [69, 112, 113]. Another important contributor is RAGE, which interacts with ligands such as HMGB1 to perpetuate inflammatory signaling, ultimately leading to tissue degeneration and impaired healing [110, 114].

As inflammation persists, fibroblasts and myofibroblasts become activated, leading to excessive collagen deposition and fibrosis formation. This fibrotic response is primarily driven by TGF-*β*1 and NF-*κ*B, both of which upregulate the expression of fibrogenic markers [113, 115]. The sustained inflammatory environment further disrupts normal repair mechanisms, resulting in ECM disorganization and weakened mechanical integrity of the tendon. Additionally, senescence of tendon-derived stem/progenitor cells, which is associated with NF-*κ*B activation, may further impair regenerative capacity [69].

While these molecular mechanisms contribute to chronic inflammation and fibrosis, they also present potential therapeutic targets. Strategies such as NF-*κ*B inhibitors and RAGE antagonists have shown promise in mitigating inflammation and promoting tendon healing. Moreover, systemic factors like smoking, which is known to exacerbate inflammation and fibrosis, should be considered in treatment approaches to optimize tendon recovery [115].

## 7. Therapeutic Implications and Future Directions

### 7.1. Targeting Inflammation in Rotator Cuff Repair

Regulating immune cell behavior, particularly macrophage polarization, is a critical approach to improving rotator cuff repair. Research indicates that biologic adjuvants can shift macrophage polarization toward the anti-inflammatory M2 phenotype while simultaneously reducing the pro-inflammatory M1 response, thereby optimizing the healing process in rotator cuff tears [116]. Low-intensity pulsed ultrasound (LIPUS) has been shown to enhance M2 macrophage polarization, accelerating the repair of the tendon–bone interface (TBI). This technique not only increases bone volume and maturity but also strengthens its biomechanical properties, such as deformation resistance and ultimate breaking strength, offering a noninvasive method to improve repair outcomes [117]. Additionally, mechanical stimulation of tissues, especially through activation of the IL-4/JAK/STAT signaling pathway, further promotes M2 macrophage polarization, improving the quality of tendon–bone integration under experimental conditions [118].

Recent advancements in MSC [99] therapies for tendon regeneration, particularly in rotator cuff injuries, have shown promising developments. One significant innovation involves the use of kartogenin-loaded exosomes derived from bone marrow mesenchymal stem cells (Kl-BMSC-Exos), which have demonstrated enhanced chondrogenesis and fibrocartilage regeneration in a rat model of rotator cuff injury. This approach improves the biomechanical properties of the tendon enthesis, potentially reducing retearing rates postsurgery by promoting organized collagen fiber arrangement and upregulating Mospd1, a gene associated with fibrocartilage regeneration [119]. Additionally, clinical trials are exploring the efficacy of microfragmented adipose tissue implantation, which may improve pain and functional outcomes compared to conventional surgery, suggesting a potential for standardization in clinical practice [120]. The literature also highlights the potential of MSCs from various sources, such as adipose tissue, to reduce inflammation, enhance tissue remodeling, and improve tendon strength, although human trials have yielded mixed results, underscoring the need for more standardized investigations [121]. Furthermore, three-dimensional cultures of rotator cuff-derived MSCs have been shown to exhibit superior regenerative capabilities compared to two-dimensional cultures, indicating a promising direction for enhancing MSC therapy efficacy [122]. Overall, while MSC therapies offer a promising alternative to traditional treatments, further rigorous clinical trials are necessary to fully establish their efficacy and safety in rotator cuff injury management [123].

Biological and genetic therapies offer additional support by targeting inflammatory processes to create a healing-conducive environment. Advanced bioactive dressings, such as those derived from umbilical cord matrices, have shown significant promise in regulating injury microenvironments by promoting cell proliferation, migration, and cartilage formation while effectively controlling inflammation. Evidence from preclinical animal studies and early human trials further supports their efficacy in enhancing recovery outcomes [124]. Similarly, mesenchymal stem cells sourced from the bone marrow and adipose tissue are increasingly recognized for their dual ability to suppress inflammation and promote tissue repair. However, their full therapeutic potential remains to be validated through rigorous clinical investigations [121].

Hydrogels enriched with growth factors like IGF-1, TGF-*β*1, and PTH have demonstrated improvements in both the biomechanical strength and histological structure of repaired tissues in preclinical rotator cuff models. These encouraging results suggest the potential for clinical translation to improve healing outcomes [125]. Meanwhile, platelet-rich plasma (PRP) and pluripotent cell populations continue to be explored for their regenerative properties, though inconsistencies in characterization and a lack of standardization have resulted in variable clinical outcomes [126]. Novel composite hydrogels that release anti-inflammatory agents like curcumin and differentiation-promoting ions such as Mg2+ have demonstrated efficacy in enhancing tendon-to-bone healing by simultaneously suppressing inflammation and promoting differentiation in preclinical models [127].

In summary, therapeutic strategies that focus on modulating inflammatory pathways through macrophage polarization, biologic adjuvants, stem cell therapies, and advanced biomaterials offer promising avenues for improving rotator cuff repair outcomes. These multifaceted approaches aim to balance inflammation and regeneration, thereby optimizing the healing microenvironment and enhancing clinical success rates.

### 7.2. Personalized Medicine Approaches

**Figure 3:**
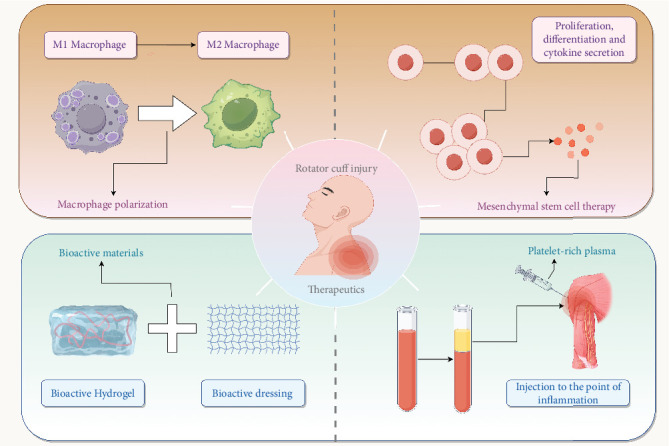
Therapeutic strategies for rotator cuff injuries. The schematic highlights key approaches for rotator cuff repair, including macrophage polarization modulation, mesenchymal stem cell therapy, bioactive dressings, hydrogels, and platelet-rich plasma (PRP). Single-cell sequencing enables precision medicine by uncovering immune dynamics and guiding personalized therapeutic strategies.

Single-cell sequencing revolutionizes the development of personalized therapies for rotator cuff injuries by providing high-resolution immune cell analysis (Figure 3). By identifying immune cell subtypes and their functional states, scRNA-seq uncovers critical insights into the immune dynamics involved in tissue repair and inflammation. For example, the TempO-LINC method enhances both the sensitivity and throughput of single-cell analysis, enabling the identification of unique cell states and molecular pathways that serve as therapeutic targets for individualized interventions [128].

High-throughput scRNA-seq methods excel in recovering gene expression signatures and identifying differentially expressed genes, unveiling the heterogeneity of immune responses in vivo [129]. These insights are particularly significant for rotator cuff injuries, where diverse immune cell populations influence inflammation, repair, or the progression to chronic pathology.

Applications of scRNA-seq in autoimmune conditions, such as juvenile idiopathic arthritis, have demonstrated its capability to characterize patient-specific immune profiles and analyze differential expression responses to stimuli [130]. This underscores its potential to dissect immune cell dynamics in rotator cuff injuries, where effective targeted interventions rely on understanding individual immune variability.

Furthermore, the integration of transcriptomic and proteomic data through multiomics single-cell sequencing offers a comprehensive perspective on immune cell characteristics and functions. This approach is critical for identifying biomarkers that underpin the development of personalized therapeutic strategies [92]. Technologies like dCODE Dextramer further advance immune profiling by enabling antigen-specific analyses of T- and B-cell responses, providing precise insights into the mechanisms driving inflammation and tissue repair [131].

In summary, single-cell sequencing technologies offer a detailed map of immune cell heterogeneity and regulation, enabling the development of precision therapies tailored to individual immune profiles. By addressing patient-specific immune dynamics, these advanced tools hold great promise for improving outcomes in rotator cuff repair [132, 133].

### 7.3. Challenges and Future Perspectives

Applying scRNA-seq to complex tissues, such as the rotator cuff, poses numerous technical challenges due to the intrinsic complexity and heterogeneity of these tissues. A primary obstacle is the difficulty in isolating individual cells from such tissues. Traditional methods often require extensive manual handling, which can result in cell loss or damage and may fail to ensure uniform cell populations due to varying dissociation conditions for different cell types [134]. Additionally, the presence of multinucleated cells, such as muscle fibers in the rotator cuff, adds to the complexity. These cells are difficult to encapsulate in individual droplets, and multiple nuclei in single-nucleus transcriptomics further complicate the process [77]. The inherent technical noise and variability of scRNA-seq can obscure the gene expression profiles of distinct cell types, making data comparison and analysis challenging [135]. Another limitation of conventional scRNA-seq is the lack of spatial context; the processes of tissue dissociation and cell isolation can lead to the loss of critical location-specific transcriptomic information, which is essential for understanding the spatial diversity of tissues like the rotator cuff [136]. Moreover, inconsistencies in RNA capture rates and platform-dependent biases present additional barriers. Certain scRNA-seq technologies may fail to adequately represent specific cell types, especially those with low mRNA levels, leading to biased transcriptomic data [137]. Furthermore, the lack of ethnic diversity in scRNA-seq datasets limits the identification of rare cell types and subpopulations, which is essential for advancing precision medicine and comprehending the intricate cellular diversity in complex tissues [138].

Incorporating scRNA-seq findings into the clinical management of rotator cuff injuries could revolutionize our understanding and treatment of these complex musculoskeletal conditions. The detailed analysis of gene expression provided by scRNA-seq is crucial for identifying distinct cell types and elucidating their roles in disease progression and repair mechanisms [74, 139]. For rotator cuff injuries, this technology can uncover the heterogeneity of involved cell types, such as synovial fibroblasts, which undergo state transitions and engage in complex intercellular communication during both chronic and acute shoulder injuries [61]. This comprehensive cellular map can guide the development of targeted therapies that address the specific cellular and molecular mechanisms underlying rotator cuff disease. Furthermore, the application of scRNA-seq in precision medicine can facilitate the identification of disease-driving genes and enable personalized treatment approaches, potentially improving outcomes for patients with rotator cuff injuries [140]. Spatial transcriptomics adds an essential dimension by illuminating localized cellular dynamics within muscle tissue, which is vital for interpreting phenotypic changes associated with rotator cuff injuries [77]. However, despite the promise of these cutting-edge techniques, challenges remain, including the need for advanced bioinformatics tools to handle complex datasets and the technical difficulties inherent in single-cell sequencing [139, 140]. Nevertheless, integrating these advanced sequencing technologies with current clinical practices, such as surgical interventions and regenerative therapies, could lead to more effective, personalized treatment strategies for rotator cuff injuries, ultimately reducing the socioeconomic burden of these conditions [141].

## 8. Conclusion

The advent of single-cell sequencing has significantly transformed our understanding of the immune microenvironment in rotator cuff injuries. It has unveiled the diverse cellular composition within tendons and surrounding tissues, as well as the intricate networks of cellular interactions. This groundbreaking research highlights the critical roles of macrophages, T-cells, fibroblasts, and various immune and nonimmune cells in regulating inflammation, tissue remodeling, and fibrosis progression. At the heart of these findings is the dynamic regulation of cytokine and chemokine networks, along with pivotal signaling pathways—such as NF-*κ*B, AMPK, and TREM-1—that guide the transition from inflammatory to healing immune responses, ultimately influencing tendon repair and pathological outcomes.

A key theme emerging from these discoveries is the dual role of inflammation in tendon healing. While acute inflammation is essential for initiating tissue repair, its chronic persistence often leads to fibrosis, degeneration, and impaired function. The interactions between immune and stromal cells, particularly macrophage–fibroblast and immune–vascular crosstalk, underscore the critical importance of cellular communication in determining tissue outcomes. By elucidating the mechanisms underlying chronic inflammation and fibrosis, single-cell technologies provide a foundation for developing targeted therapies that mitigate maladaptive responses and enhance regenerative processes.

Looking ahead, the integration of single-cell sequencing with spatial transcriptomics and multiomics approaches holds great promise for revolutionizing the management of rotator cuff injuries. These methods will deepen our understanding of tissue-specific and regional cellular dynamics, paving the way for innovative therapeutic strategies. Future research should focus on leveraging these technologies to refine personalized medicine, identify reliable biomarkers of disease progression, and optimize biologics and gene therapies. Addressing current challenges, such as technical variability, the need for spatial context, and the lack of ethnic diversity in datasets, will be crucial for maximizing the clinical applicability of these findings.

By advancing our understanding of the cellular and molecular underpinnings of rotator cuff pathology, single-cell sequencing has the potential to improve treatment outcomes, address the challenges of chronic tendon disorders, and significantly impact musculoskeletal health. Continued innovation in this field is poised to shape the future of tendon biology and regenerative medicine, offering new hope for effective, personalized care for patients with rotator cuff injuries.

## Data Availability

The data that support the findings of this study are available from the corresponding author upon reasonable request.
